# A rare case of right upper lung cancer with azygos lobe and partial anomalous pulmonary venous return

**DOI:** 10.1186/s13019-022-01823-9

**Published:** 2022-04-12

**Authors:** Xiaoyang Su, Qianzhun Huang, Zhiqiang Luo, Ning Fang, Jian Huang

**Affiliations:** Department of Thoracic Surgery, Maoming People’s Hospital, 101 Weimin Road, Maoming, 52500 China

**Keywords:** Lung cancer, Azygos lobe, Partial anomalous pulmonary venous return, Three-dimensional reconstruction

## Abstract

**Background:**

The azygos lobe (AL) combined with partial anomalous pulmonary venous return (PAPVR) is comparatively uncommon as well as in radical surgery for right lung cancer.

**Case presentation:**

We herein present an extremely rare case of lung cancer coexisting with AL and asymptomatic PAPVR, which was diagnosed with preoperative contrast three-dimensional reconstruction and received radical surgery by thoracoscopy. During the surgery, we preserved azygos vein successfully and found a split type of PAPVR in right upper pulmonary vein.

**Conclusions:**

AL combined with PAPVR may cause confusion on the vascular separation and disconnection of the right pulmonary hilar. However, preoperative 3D reconstruction is more conducive to the correct performing of this type of surgery.

## Background

The azygos lobe (AL), caused by azygos vein (AV) migrating abnormally from thoracic wall to tracheobronchial angle, is a rare variantion in the right upper lobe (RUL) [1]. Partial anomalous pulmonary venous return (PAPVR) is a less uncommon congenital pulmonary venous anomaly that involves drainage of 1 to 3 pulmonary veins into the right-sided circulation [2]. The prevalence of the AL is 1.2% in CT scans while PAPVR is only 0.1–0.2% [3]. At present, there is no report on combination of such two type malformations as well as radical surgery for right lung cancer. Herein we present the case of lung cancer coexisting with AL and asymptomatic PAPVR, which was diagnosed by preoperative contrast three-dimensional reconstruction (3D- reconstruction) and the patient received radical surgery. During the surgery, we preserved AV successfully, and avoided massive hemorrhage. A split type of PAPVR, which is often confused with right middle pulmonary vein (RMV), was found in right upper pulmonary vein (RUV). Therefore, we have summarized prior experience regarding the aforementioned vascular malformations.

## Case presentation

**Fig. 1:**
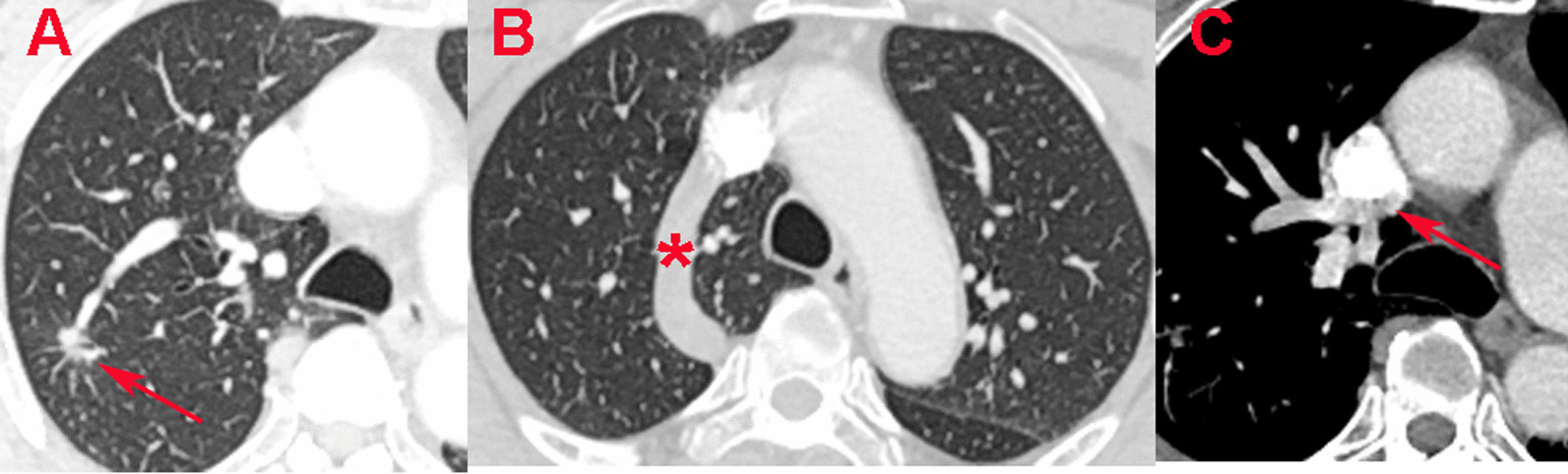
**a** The malignant nodule in in two-dimensional CT scans; **b** asterisk represents the dilated azygos vein; **c** The red arrow represents partial anomalous pulmonary venous return (PAPVR) drainages into superior vena cava

A 60-year-old male patient was admitted to our department due to incidental observation of a 10-mm partly solid nodule with spiculated sign and boundary unclear in RUL by annual physical CT scans (Fig. 1a). The tumour markers were elevated and malignant nodule was highly suspected, with squamous cell carcinoma antigen 1.70 ng/mL (reference 0–1.5 ng/mL), CYFRA21-1 3.99 ng/ml (reference 0–2.1 ng/mL). Pulmonary function tests were appropriate for lung resection; FVC 4.76L (Pred 131.4%), FEV1 3.89 L (Pred 97.4%) and the diffusion function (MVV) 164.97L/min (Pred 97.4%) without significant abnormalities of echocardiography. Contrast CT described abnormal courses of AV and separated the AL from RUL (Fig. 1b). Surprisingly, it is found that part of the pulmonary veins converged directly into the superior vena cava (Fig. 1c). Therefore, a preoperative 3D-reconstruction was performed which revealed AL combined a split type of PAPVR in RUV.

**Fig. 2:**
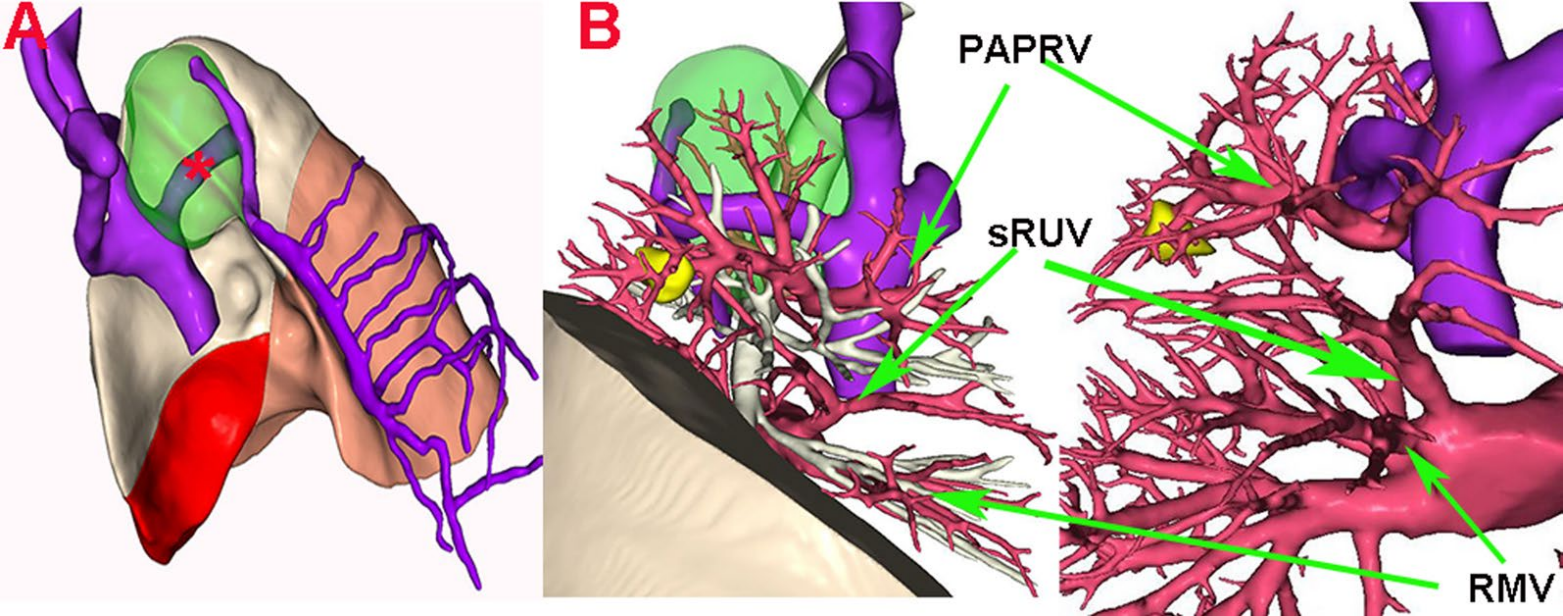
Three-dimensional reconstruction of computed tomography. **a** Asterisk represents the dilated azygos vein(AV), the green part of the lung is azygos lobe (AL). **b** PAPVR, partial anomalous pulmonary venous return; sRUV, small branch of right upper pulmonary vein; RMV, right middle pulmonary vein, and the nodule in yellow

The RUV was divided into two branches with different diameters (Fig. 2). The thicker branch drained most of the anterior and atypical veins as well as part of the posterior veins (V1 + V3ac + V2b), which was identified as PAPVR with AV into the superior vena cava, while the other small branch (V2ac + V3b, represented by the below sRUV) drained into the left atrium at the proximal end of RMV.

**Fig. 3:**
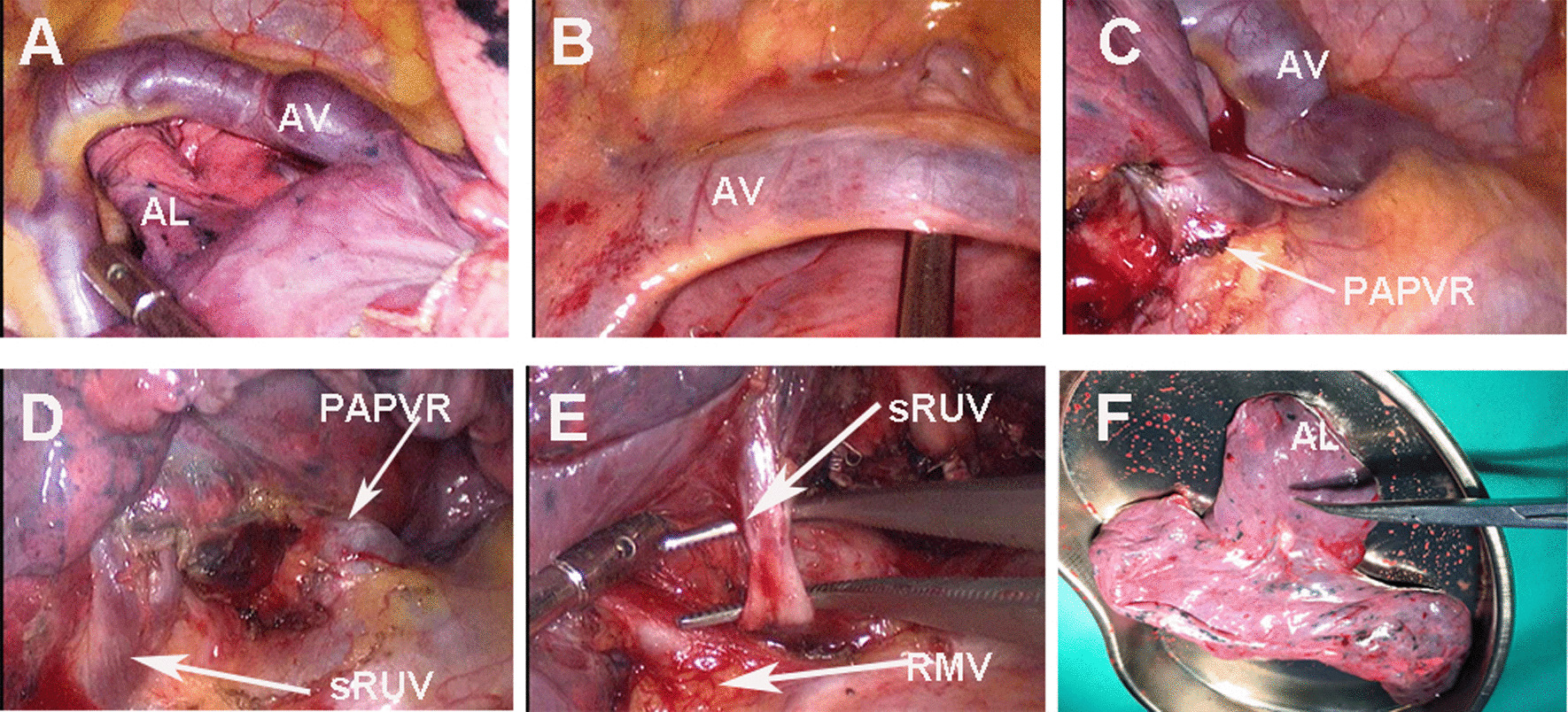
Intraoperative findings: **a** dilated azygos vein (AV) and azygos lobe (AL), **b** The cavity formed by invaginated parietal and visceral pleura with AV. **c** Partial anomalous pulmonary venous return (PAPVR) and AV drainage into superior vena cava. **d**, **e** The splited tppe of right upper pulmonary vein (RUV), sRUV, small branch of RUV; RMV, right middle pulmonary vein. **f** The resected right upper lobe combined AL shaped like a clove

After the exclusion of distant metastasis, two-port thoracoscopic right upper lobectomy and mediastinal lymph node dissection were performed. During the surgery, the abnormally dilated AV connected to the superior vena cava, and crossed the apex of the lung from the ventral to the dorsal side (Fig. 3a). When the RUL was pulled down to the caudal side, the AL could be slipped out from the cavity consist of AV and pleuras smoothly (Fig. 3b). In the hilar, the thicker RUV branch draining into the superior vena cava was confirmed (Fig. 3c). After sutured the PAPVR of RUV by stapler, the pulmonary artery and bronchi of RUL were exposed as normal. For the hypoplastic of the fissure between the middle and upper lobe, we carefully separated sRUV, the veins of middle and lower lobe on mediastinal side. Under the premise of the RMV safety confirmed, the sRUV was ligated with silk thread and the fissure was separated by stapler. Shaped like a clover, the resected RUL combined AL was consistent with the 3D- reconstruction (Fig. 3f).

The operation lasted 285 min with 50 ml blood loss. The postoperative course was uneventful. The drainage tube was removed on the Day 3 and the patient was discharged on the 10th postoperative day in good clinical condition. Pathological analysis showed lung adenocarcinoma without lymph nodes involved.

## Discussion and conclusion

AL combined with PAPVR is a relatively rare variation in RUL, which is usually diagnosed incidentally on imaging in asymptomatic. The AL results from a failure of the normal migration of the AV from the thoracic wall to its usual position at the tracheobronchial angle, such that the fissure of the parietal and visceral pleura remain invaginated, which forming a cavity with the AV at its base [1, 4]. Furthermore, the potential cavity would not cause adhesion to the AL [5]. PAPVR is often associated with hypoplastic right lung and congenital heart disease, such as atrial septal defect, resulting in a left-to-right shunt. The patient’s clinical severity is determined by the degree of the left-to-right shunt [6]. Inadequate recognition of AL may easily injury AV leading to mass hemorrhage [4]. Lobectomy is the definitive treatment for lung cancer and PAPVR, and no hemodynamic problems occurred during and after the operation [7]. However, if PAPVR is located in different lobes and preserved wrongfully, inadequate resection may increase the left-to-right shunt flow and cause right-sided heart failure, increasing the risk of postoperative mortality [8].

In this case, AL was discovered accidentally with asymptomatic. To understand the variations of mediastinal vascular, we performed preoperative 3D reconstruction. The split RUV was found unexpectedly and partially as PAPVR. However the patient had no abnormalities of preoperative echocardiography without symptomatic, for the small degree of the left-to-right shunt. Intraoperatively, we found that the azygos fissure did not cause adhesions of AL. Hypoplastic of the fissure between the middle and upper lobe, the thicker branche of RUV identified as PAPVR may be misjudged as intact RUV, while the split small branche (sRUV) was confused with RMV (Fig. 3e), resulting in incomplete resection of RUV. Moreover, in this case, the PAPVR was mainly located in the RUV and lobectomy did not increase the flow of left-to-right shunt.

To sum up, if AL is detected prior to surgery, it should be aware that there may be other vascular variations. Therefore, contrast CT completion and vascular reconstruction might be a desirable option for AL surgery. The appearance of AL would not increase the difficulty in lobectomy, and AV could be preserved successfully. As for such vascular malformations as PAPVR, echocardiography should be performed to exclude congenital heart disease. Being familiar with anatomy of PAPVR is good for preventing accidental damage to the vessel, and especially when split type of RUV combined with hypoplasia fissure. Indeed, preoperative 3D reconstruction, which is safe and fast, is more conducive to the correct performing of this type surgery, for instance, the identification of small branch of RUV and preservation of RMV..

## Data Availability

Not applicable.

## Abbreviations

AL: Azygos lobe
AV: Azygos vein
PAPVR: Partial anomalous pulmonary venous return
RUL: Right upper lobe
RUV: Right upper pulmonary vein
RMV: Right middle pulmonary vein
